# Ethical competence in DNR decisions –a qualitative study of Swedish physicians and nurses working in hematology and oncology care

**DOI:** 10.1186/s12910-018-0300-7

**Published:** 2018-06-19

**Authors:** Mona Pettersson, Mariann Hedström, Anna T. Höglund

**Affiliations:** Department of Public Health and Caring Sciences, Box 564, 751 22 Uppsala, Sweden

**Keywords:** Ethical competence, DNR decisions, Oncology, Hematology, Nurses, Physicians

## Abstract

**Background:**

DNR decisions are frequently made in oncology and hematology care and physicians and nurses may face related ethical dilemmas. Ethics is considered a basic competence in health care and can be understood as a capacity to handle a task that involves an ethical dilemma in an adequate, ethically responsible manner. One model of ethical competence for healthcare staff includes three main aspects: *being, doing* and *knowing,* suggesting that ethical competence requires abilities of character, action and knowledge. Ethical competence can be developed through experience, communication and education, and a supportive environment is necessary for maintaining a high ethical competence. The aim of the present study was to investigate how nurses and physicians in oncology and hematology care understand the concept of ethical competence in order to make, or be involved in, DNR decisions and how such skills can be learned and developed. A further aim was to investigate the role of guidelines in relation to the development of ethical competence in DNR decisions.

**Methods:**

Individual interviews were conducted with fifteen nurses and sixteen physicians. The interviews were analyzed using thematic content analysis.

**Results:**

Physicians and nurses in the study reflected on their ethical competence in relation to DNR decisions, on what it should comprise and how it could be developed. The ethical competence described by the respondents related to the concepts *being, doing* and *knowing*.

**Conclusions:**

In order to make ethically sound DNR decisions in oncology and hematology care, physicians and nurses need to develop appropriate virtues, improve their knowledge of ethical theories and relevant clinical guidelines. Ethical competence also includes the ability to act upon ethical judgements. Continued ethical education and discussions for further development of a common ethical language and a good ethical working climate can improve ethical competence and help nurses and physicians cooperate better with regard to patients in relation to DNR decisions, in their efforts to act in the best interest of the patient.

## Background

In almost every kind of care, patients can sometimes be considered to have such a poor prognosis that they would not survive cardiopulmonary resuscitation (CPR) for cardiac arrest, or would survive with poor function and quality of life. A do-not-resuscitate (DNR) order can then be made by the responsible physician. The meaning of DNR is that neither basic (heart compressions and ventilation) nor advanced (defibrillator or medicines) CPR should be performed. If the patient does not have a DNR order, CPR must start within 60 s, according to Swedish guidelines [1].

In oncology and hematology care, decisions on DNR are made regularly, but the context of these decisions can differ between the specialties. In oncology, a patient with metastases can be incurable, but still have a long time left to live with a good quality of life, and in these cases the palliative phase can thus be quite long. In hematology, on the other hand, a patient can be severely and life-threateningly ill due to treatment, but remain in the curable phase until all available treatments have been given. In those cases the palliative phase can be short, sometimes only a few days. Due to the severity, and often also stigmatization, of a cancer diagnosis, and the occasionally long curative treatment periods, patients and their families might be vulnerable in these situations. This imposes major ethical demands on making decisions regarding DNR, and the information given must be clear and adapted to the situation at hand.

Our previous research has revealed ethical dilemmas, which physicians and nurses may face in relation to DNR decisions in oncology and hematology care [2]. Ethical dilemmas are situations in which a person must choose between actions that are apprehended as equally correct ethically. The choice renders one action deselected, and it is therefore impossible to perform all ethically important actions [3–5]. Ågren Bolmsjö et al. [6] describe an ethical dilemma as a situation in which there is a choice between different actions, and it is impossible to fulfill all parties’ interests in a way that satisfies everyone.

Examples of ethical dilemmas in DNR decisions in oncology and hematology care as revealed by our previous studies include: disagreement in the team regarding whether a patient should have a DNR order or not; when patients and relatives think differently about DNR; when a choice of whether or not to implement a DNR order stands between patient autonomy and the patient’s medical prognosis; and when the patient and family have not been informed of the DNR order by the physician and ask the nurse about what has been decided [2].

When ethical dilemmas occur, different values, norms or interests must be weighed against each other. Different models have been developed for such moral judgements and there are well-established examples of theories that judge ethical dilemmas based on *consequences* (e.g. *utilitarianism*, where the goal is to maximize the good consequences for as many individuals as possible) or *duties/rights* (so-called *deontological theories*, where an action is regarded as right or wrong in itself, irrespective of the consequences, such as telling the truth or not harming others). These traditions are mirrored in the four well-known ethical principles of *autonomy, non-maleficence, beneficence* and *justice* [7]*.* The principles of autonomy and justice are derived from deontological reasoning, meaning that we have a duty to respect human dignity in every person and treat everyone as equals, regardless of consequences. The principles of non-maleficence and beneficence are utilitarian in character, as they prescribe maximizing the well-being of others by promoting good consequences and limiting harm. Apart from utilitarianism and deontology the *ethics of virtue* is another well-established theory in medical ethics. Here, the character of the agent is at the fore. A person is virtuous in that s/he develops certain characteristics. Thereby, s/he can develop a suitable manner of action for a certain context or practice. A basic assumption is that a ‘good’ person performs ‘right’ actions [8–11].

As ethics is considered a basic competence in health care, ethics is part of the curriculum in the training for both nurses and physicians in Swedish education system. Since training for physicians is longer than for nurses, the amount of ethics education is a bit larger for physicians than for nurses. Further, ethical guidelines have been developed for different staff categories, e.g., the International Council of Nurses (ICN) Code of Ethics [12] and the International Code of Medical Ethics for Physicians [13]. But ethical guidelines have also been developed in relation to certain diagnoses or decisions, for example DNR. In Sweden, the Swedish Resuscitation Council, the Swedish Society of Medicine and the Swedish Society of Nursing released common ethical guidelines for CPR in 2013 [14]. These guidelines state, among other things, that there is no ethical difference between refraining from CPR and starting CPR and then withholding it. The guidelines also state that, concerning the claim for information and patient consent, there may be situations in which a patient and/or a significant other may suffer more as a result of being informed of a DNR decision than if they had not been informed. As guidelines, they are advisory, unlike laws, which are compulsory.

### Theoretical framework: ethical competence

It is reasonable to argue that since ethical dilemmas do occur in relation to DNR decisions in oncology and hematology care, staff involved in these decisions need adequate competence to handle these dilemmas. In the literature, such competence is often referred to as *ethical competence*. In short, ethical competence can be defined as a capacity to handle a task that involves an ethical dilemma in an adequate, ethically responsible manner [15].

The European Commission [16] describes ethical competence as a “meta competence,” i.e., as an integral part of knowledge, abilities and skills. The Commission stresses that such competence is essential for the development of responsibility and autonomy in an individual. Ethical competence has been studied in settings other than health care, for example in research with possible dual use [17] and in management [15]. In health care the concept has been defined in many ways, and as Kulju et al. [18] state, no consensus on the definition can be found in the literature. In their conceptual analysis of ethical competence, based on 18 articles (12 theoretical and 6 empirical), they propose the following common “attributes” for ethical competence: *strength of character, ethical awareness, moral judgment* and *the will to do good*. In the review of Lechasseur et al. [19] the following components of ethical competence in nursing practice were suggested: *ethical sensitivity, ethical knowledge, ethical reflection, ethical decision-making, ethical action* and *ethical behavior.*

A slightly different approach is suggested by Eriksson et al. [20]. Their model of ethical competence for healthcare staff includes three main aspects: *being, doing* and *knowing*. In short, this means that ethical competence requires abilities of character, action and knowledge. Character, or in Eriksson’s et al. [20] words, the ethics of *being*, can be understood as a form of virtue ethics, which concerns good character traits in a person. The ethics of doing, on the other hand, are concerned with how we should act in ethically challenging situations. Models of weighing consequences or following duties, as for example in utilitarianism and deontology, are typical examples of such an ethics of *doing*, since they are primarily concerned with how to act. However, Eriksson et al. [20] suggest that ethical competence must also include *knowing*, i.e., knowledge of ethical theories, such as deontology and utilitarianism, but also of relevant ethical guidelines. A communicative model is suggested in which the work organization should provide opportunities for regular ethical discussions in which all aspects of ethical competence can be alerted. In this article, the model suggested by Eriksson et al. [20] will function as the theoretical framework, since their suggestion includes many of the elements identified by both Kulju et al. [18] and Lechasseur et al. [19].

Apart from striving to capture the meaning of the concept “ethical competence,” previous research has also studied how such competence can be developed and learned. Kulju et al. [18] suggest that ethical competence can be developed through experience, communication and education. Furthermore, they emphasize the importance of a supportive environment and organization. Similar approaches are found in the studies by Falkenström et al. [15], Eriksson et al. [20] and Höglund et al. [21]. Research has also suggested that in order to develop and maintain a high level of ethical competence in health care, ethical deliberation, in the form of regular ethical discussion forums, is required [22].

In sum, one can state that decisions on DNR are frequently made in oncology and hematology care and that ethical dilemmas are likely to arise in relation to these decisions. Since ethical competence is crucial for dealing with ethical dilemmas, it is important to investigate the caregivers’ conceptions of ethical competence in this context, how it can be learned, developed, and how it is used in clinical practice.

### Aim

The aim of the present study was to investigate how nurses and physicians in oncology and hematology care understand the concept of ethical competence in order to make, or be involved in, DNR decisions and how such skills can be learned and developed. A further aim was to investigate the role of guidelines in relation to the development of ethical competence in DNR decisions.

## Method

### Sample and participants

The study was conducted in hematology and oncology departments in central Sweden. Fifteen nurses from four hospitals and sixteen physicians from seven hospitals participated. Nursing unit managers and heads of the departments introduced the study to eligible nurses and physicians and asked for interest in participation. The first author contacted some of the suggested participants and was contacted by some. All individuals with whom initial contact was taken were included in the study, except one nurse, who was then replaced by another nurse from the same department. After receiving written and oral information, all respondents agreed to participation by signing an informed consent form. Characteristics of the participants are presented in Table 1.

**Table 1:** Demographic characteristics for participants

Variables	Nurses *n* = 15	Physicians *n* = 16
Gender		
Female	12	8
Male	3	8
Age (years)		
21–30	9	1
31–40	5	3
41–50	–	5
51–60	1	5
61–70	–	2
Experience as a nurse/physician		
1–2 years	5	–
3–5 years	5	1
6–10 years	3	3
10–15 years	1	–
16–20 years	–	4
21–25 years	–	2
26–30 years	1	1
31–35 years	–	4
36–40 years	–	1

### Data collection

Interviews were conducted with nurses and physicians who work in oncology and/or hematology. Portions of the data, collected through interviews, have been published previously [2]. While those studies focused on the clinical parts of perceptions on and experiences with DNR decisions, the data on ethical competence and guidelines were saved to be presented separately. Hence, the main topics of the interviews that are presented in this paper are: *Knowledge of guidelines, Content of guidelines, Understanding of ethical competence,* and *Need for ethical competence in DNR decisions*. Some nurses mentioned the development of ethical competence during their interviews. Thus, the topic *Development of ethical competence* was added to the interviews with the physicians.

All interviews were performed by the first author and lasted between 23 and 67 min. They took place in rooms close to the wards at the hospitals, or in some cases in the physicians’ offices. The interviews were recorded and transcribed verbatim.

### Analysis

The transcribed interviews were analyzed using thematic content analysis [23]. The process was initially done by the first author, who read the transcripts and made notes in the margins as a first open coding. In the second stage, all margin notes were listed. The list was examined for overlaps and similarities, and used for sorting the meaning units under appropriate codes, creating subcategories which were grouped together in categories and a theme (Table 2). The co-authors listened to random selections of recordings, read selections of transcripts and participated in the analysis process, including creating categories. The final version of the analysis was approved in consensus with all authors.

**Table 2:** Description of the analysis process

Interview transcript	Initial coding framework	Final coding framework		
		Subcategory	Category	Theme
*“Sometimes I think the information requirement is a little too rigid. I know that in practice, it isn’t followed. And I can therefore find it unnecessary, because it places blame on health professionals for not discussing, when they have actually tried to be humane and compassionate in the situation.”*	The request for information is perceived as rigid and can produce unnecessary guilt if not followed.	Obstacle	The role of guidelines	Ethics in relation to DNR decisions

### Ethical considerations

The research followed international guidelines for empirical research, as outlined in the Helsinki Declaration [24], and national regulations and guidelines [25, 26]. According to Swedish legislation [26], no approval from the Regional Ethical Review Board was needed for the study. Permission for the interviews for each study was given by the head of the departments. Each participant received written and verbal information before signing the informed consent form, including information on voluntary participation, on the fact that the data would be kept confidential, and that they could terminate their participation at any time.

#### Rigor of the study

In order to enhance dependability, the interview guide for each group of respondents was the same. One researcher performed all interviews with both groups [27]. Since the participants had no difficulty understanding and answering the questions during the interviews, credibility was strengthened [28]. The interviewer also confirmed the respondents’ answers, asking for clarifications and using probing questions when needed [28].

As a nurse who has worked in the same specialty as the respondents, the interviewer was aware that her pre-understanding could be a bias. This awareness enhanced confirmability [28]. The credibility was further strengthened by the fact that all co-authors cooperated on the analysis process, through identifying and formulating the theme, categories and subcategories. The processing and quotation selections for each category/subcategory were made in consensus [27].

## Results

**Fig. 1:**
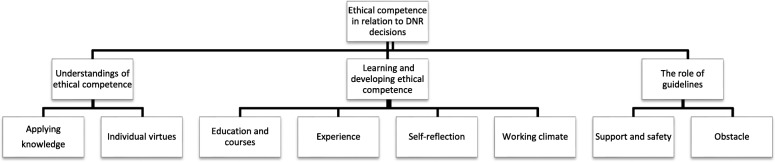
Description of theme, categories and subcategories

The analysis resulted in an overall theme, related to the aim and the interview questions: *Ethical competence in relation to DNR decisions*. Under this theme, several subcategories were grouped together into three categories: *Understandings of ethical competence, Learning and developing ethical competence,* and *The role of guidelines.* Theme, categories and subcategories are presented in Fig. 1. In the following sections, each category is illustrated by quotes. The sign [...] indicates excluded insignificant statements.

### Ethical competence in relation to DNR decisions

#### Understandings of ethical competence

The respondents’ understandings of ethical competence resulted in two subcategories. *Applying knowledge* describes the importance of formal medical knowledge related to the context, as well as ethical competence comprising the ability to apply ethical models, to weigh ethical values against each other and to be aware of different perspectives in a situation. The *Individual virtues* subcategory highlights the respondents’ views of the importance of individual character traits in the caregiver.

##### Applying knowledge

Several respondents emphasized that ethical competence was always necessary in their work, not only in DNR or other decisions concerning prolonging life or not. However, several physicians expressed that medical knowledge was a prerequisite for ethical competence regarding DNR decisions. Without thorough knowledge about the patients’ diseases and conditions, the physician might be incapable of making the right ethical decisions, as these must be based on correct medical judgements.

*“Basically, I think you need formal knowledge. You can’t make an ethical decision if you don’t have formal knowledge as a foundation. I can’t make decisions on complex neurological patients, an ALS patient for example, if I don’t have knowledge of the disease.” Physician # 8*

All respondents, both nurses and physicians, mentioned several aspects of ethical competence which they regarded as necessary for making or participating in DNR decisions.

*“You definitely need ethical skills ... You have to be interested in the person you have in front of you and understand that person. And you need to have medical skills, to know ... what you can do for this patient medically ... That is also a kind of ethics. You can’t make an ethical decision of this kind without being firmly grounded in science ... If so, you’re in trouble.” Physician # 12*

The respondents also mentioned the importance of knowledge about ethics and the ability to identify value conflicts as a part of ethical competence. Some of the ethical values the respondents mentioned were primarily directed at the patient, such as providing the best possible care to the right patient; seeing and understanding the person as well as the patient; and respecting patient integrity. They also mentioned the importance of seeing patients as the different individuals they are.

*“To respect ... that everyone is different even though they might have the same diagnosis ... it’s different from person to person. There can be two people who look the same and have the same diagnosis, but it can still be different in so many ways. In one case it might be ethically right to do this and in another case it can be ethically right to do that, because people are different.” Nurse # 14*

Other aspects mentioned as components of ethical competence were knowledge of ethical theories and principles, such as utilitarianism and the principle of human dignity.

*“You can take a utilitarian perspective – the greatest happiness to the greatest number of people – or you can reason according to the principle of human dignity. If your judgement is based on the pursuit of happiness among the majority, then you might not care for those who are seriously ill or dying, and those who have challenging diseases that might be very expensive to treat, because it doesn’t benefit the majority. It would be good for one person, but not for others. But if you base your argument on the principle of human dignity, then it is the right thing to do, even though it doesn’t create happiness for everyone, and even though it might be costly. So, which principle are we to support?” Physician # 11*

*“Well, there are different criteria ... with regard to human dignity and other principles. They are all pretty good, because ... they make you aware of things [...]. You might have prejudices and so on ... But if you think ethically … I believe you can reason in another way.” Nurse # 15*

Some respondents described ethical competence as the ability to weigh ethical values against each other, e.g., weighing patient autonomy against not causing harm, or patient autonomy versus doing good, and non-maleficence versus beneficence.

*“What am I doing and why am I doing it? Can this cause harm, or can this be beneficial? And how great is the harm and how great are the benefits?” Physician # 8*

*“To be able to argue for what is reasonable, maybe. Am I causing suffering now? Things like that. The patient’s autonomy and integrity, and the relatives’… To, in some way, be able to weigh arguments against each other.” Nurse # 13*

Ethical competence was also expressed as perceiving where in the process the patient and family members were, and being able to meet them on their different levels. Both physicians and nurses mentioned the value of listening and being able to respect different opinions and interests.

*“To think about what we are doing, the meaning of what we do, how it affects others. Also, how they perceive the situation in end of life, both patients and family members.” Physician # 10*

*“A wider and more transparent way of thinking, bringing in more perspectives than you would have done otherwise.” Nurse # 15*

##### Individual virtues

In addition to referring to ethical competence as knowledge of ethics, the respondents also referred to ethical competence as characteristics in the individual caregiver. In line with the theoretical perspective described above, this could be described as a form of virtue ethics. The respondents expressed how virtues could make the caregiver a better person and also a better physician or nurse. The virtues were described as character traits, such as empathy, respect, compassion, openness, courage and humbleness. A virtue such as humbleness could also describe the respondents’ attitude toward their own ability to handle difficult situations. Some physicians expressed humbleness with regard to being the one who made the decision.

*“Respect and humbleness. And knowledge. To see the person as well as the patient. And humbleness regarding one’s own ability to understand the situation.” Physician # 1*

*“To have compassion for your fellow humans ...” Nurse # 10*

The respondents also expressed the need for the ability to act in a good and virtuous way, for example, to be caring and to show empathy.

*“Empathy and sensitivity and so on, I think that’s important [...]. Somehow, you have to give the patient and the family the impression that you really care. I believe that’s very important.” Physician # 3*

The ability to understand the situation was also mentioned as a quality, as were other skills, such as being able to communicate well and to shift your perspective.

*“You need to be able to put yourself in the position of relatives and patients when discussing and communicating what you believe.” Physician # 8*

#### Learning and developing ethical competence

The second main category, *Learning and developing ethical competence,* was divided into four subcategories: *Education and courses*, *Experience, Self-reflection* and *Working climate*.

##### Education and courses

The main part of theoretical education in ethics was provided during the physician or nurse’s training. Some physicians expressed a need for more ethics classes during their education in order to better prepare the students for clinical practice.

*“You have a golden opportunity, the greatest chance to learn during your education. Yet, it’s difficult; it’s only when faced with a patient in clinical care that you ... get personal experience with it.” Physician # 2*

##### Experience

Several respondents also expressed that ethics cannot only be taught theoretically, and that education does not make difficult situations easier to handle. They expressed that the best way to learn and develop ethical competence was by being supervised by an experienced colleague, and to learn by participating when that individual made decisions. The experienced colleague could thereby function as a role model.

*“You need to participate a few times and see how it works, so it’s not just something you suddenly ... But you should be part of the decision-making process, which is why the team is important. The younger colleague should participate and hear the reasoning of a senior colleague who is prepared to make the decision, because that’s not something you should do in the beginning.” Physician # 3*

The physicians considered time and practice to be important conditions for learning ethical skills, along with obtaining practical ethical competence by working with patients, learning from colleagues and increasing their level of experience.

*“I don’t think you can take a course and learn this. I think you need time and work experience.” Physician # 7*

##### Self-reflection

Questioning oneself and one’s decisions in self-reflection was also considered a way to improve ethical competence, according to the respondents. Several physicians described their efforts to reflect on their decisions, to have a discussion with themselves on what to do and why, how their decisions might be perceived by others, and how they might affect others. Self-reflection also required the virtue of courage, which was needed to raise the question of DNR decisions, as well as the courage to admit to making less successful decisions.

*“Well, by daring to raise these issues and say them aloud, to talk to each other and to yourself once you have made a decision, to sense whether the decision was right or wrong, and also to have the courage to admit when things go wrong – that’s probably the only way to grow.” Physician # 12*

##### Working climate

Several respondents mentioned the importance of a good climate for ethical discussions.

“*On the one hand, you’ve been discussing a lot during your education and then … just that we continue to discuss in the group of physicians. I think that’s the most important thing.” Physician # 4*

*“I think you develop your ethical competence through working and through discussions among colleagues. You need a climate in which you can raise ethically difficult issues ... not just DNR decisions, but other things as well ... and where everyone has the opportunity to debate and argue and think and talk.” Physician # 7*

Some respondents also mentioned that the resources for theoretical ethical training in the department were not continuous, but dependent on economic restrictions, and were currently less frequent than before.

*“The first years I worked here, we had an ethical forum and even a small ethics course for all nurses and physicians, with invited speakers and group discussions. That was great. But we can’t afford that any longer. But we’ve had some of those little activities with a lecture and some discussions. Well ... you know how health care is nowadays; it’s like no one really has time or energy, and no one has any money. But of course it would be desirable to have more recurrent courses ... One Ethics Day per semester.” Physician # 12*

Ethics rounds and ethical discussions were also mentioned as ways to communicate experience and patient situations, and to hear and understand others’ perspectives. But ethics discussions were more often a single event, such as a debriefing after an incident, which left the medical team frustrated.

*“Recently … we have ethics meetings quite often after a patient has passed away. But those ethics meetings might be needed before the patient dies. But now you have an ethics meeting when the patient has passed away, because everybody is wondering: ‘What was your thought process?’” Nurse # 4*

#### The role of guidelines

The category on the role of guidelines was divided into two subcategories. The guidelines were seen as providing *support and safety*, and also as an *obstacle* to acting ethically in DNR situations. DNR decision-making is an area to which the guidelines are applicable, and they thus *played an active role among the respondents. The most relevant areas were the guidelines for who makes the DNR decision, and for informing patients and relatives of the transition from curative to palliative care and the DNR decision.*

##### Support and safety

Several nurses mentioned that guidelines could contribute to everyone having the same definition of DNR and could thus make DNR decisions clearer. Some nurses expressed a need for more detailed guidelines, developed for the specialty in which they work. Such guidelines were supposed to provide support to the nurses when they felt it was time to raise the question of DNR with the physician. Such guidelines could also support nurses who might sometimes want to question a physician’s decision.

*“Then you might ... feel that you have more support, if you think a decision is wrong. You would be able to pick up the guidelines and make certain points clear ... where it actually matches, or not.” Nurse 15*

During the interviews, none of the nurses mentioned any regulation or guideline by name, or any authority with responsibility for regulating the guidelines. However, all interviewed nurses knew it was the physicians’ responsibility to make the decision and to inform patients and/or relatives, and that there were regulations around documentation of DNR decisions.

All interviewed physicians, on the other hand, were aware of the national regulations on DNR decisions. Some also mentioned local guidelines with instructions on medications and who to inform.

*“We have local procedures for palliative care, which include information on what medications should be available, and on how you should have a meeting with the family about the transition to palliative care. So there are – to some extent – written procedures.” Physician # 5*

Several of the physicians expressed that the guidelines were just advisory, not compulsory, but that they gave guidance and evidence, and a more scientific perspective on the question of DNR for different groups of patients.

*“Of course, all such guidelines are just guidance; they are advisory, but they could improve evidence-based care and provide us with a more scientific approach.” Physician # 9*

##### Obstacle

However, the guidelines could also occasionally be an obstacle rather than supportive, according to the respondents. They could hinder physicians from acting ethically regarding informing patients and relatives of the DNR decision. Several physicians said they did not always inform patients and families of DNR decisions. When they refrained from providing information about DNR decisions, they did so after ethical consideration, weighing the patient’s legal right to be informed against the risk of doing harm to this patient or the family.

*“Sometimes I think the information requirement is a little too rigid. I know that in practice, it isn’t followed. And I can therefore find it unnecessary, because it places blame on health professionals for not discussing, when they have actually tried to be humane and compassionate in the situation.” Physician # 13*

Because the guidelines were occasionally perceived as rigid, some of the physicians felt they were doing something wrong by deviating from them, while they were actually trying to meet the needs of an individual patient and to make an ethically informed decision.

*“I can fill in a form without talking to the patient and then I sometimes feel like a villain, but at the same time, I feel that I can take that. I’m doing this because I think it’s the most correct choice in this situation ... but it’s actually illegal! We may not resolve that in the moment, but I feel a little bad sometimes, and I think … maybe the requirement should not be so strict.” Physician # 12*

However, most of the physicians who did not inform patients and families about DNR decisions found other strategies for providing information about further treatment. For example, they did not use the word “DNR” but chose other words instead when explaining the future care of the patient.

*“I would rather ... tell them that the plan for the treatment of your illness looks like this and so on, and if you get much worse, we have no plans to move you to the intensive care unit, because we do not think you will benefit from that. That kind of information can be enough, and then I may write DNR in the form, but I never talk about banging on the chest or ... or I very rarely do that.” Physician # 12*

## Discussion

The main aim of this study was to investigate how nurses and physicians in oncology and hematology care understood the concept of *ethical competence* in relation to DNR decisions and how such competence can be learned and developed. A further aim was to investigate the role of guidelines in relation to the development of ethical competence in DNR decisions. The results showed that both physicians and nurses were able to reflect on their ethical competence in relation to DNR decisions, as well as on what it should comprise. If seen through the lens of the theoretical framework of this paper, the ethical competence described by the respondents can be understood in light of the concepts *being, doing* and *knowing*.

Several physicians in this study mentioned the necessity of medical competence in order to have ethical competence. Without medical knowledge of relevant diseases, it was not considered possible to make an informed decision about DNR. This is in line with previous research. Falkenström et al. [15] found that a contextual understanding is important for being able to handle ethical conflicts. Kulju et al. [18] found in their review that the experience in a profession is a prerequisite for ethical competence. Robichaux [29] emphasizes that it is important for nurses to have knowledge of pathophysiology and medical and nursing interventions in order to make good ethical or medical decisions. This might represent the medical knowledge that, according to the respondents in this study, is a necessary aspect of ethical competence. Nurses did not mention medical knowledge to the same extent, which can be related to their different work tasks. They are not responsible for making DNR decisions, but rather to provide good nursing care to the patients.

The participants in the present study also mentioned some virtues when asked about the content of ethical competence. Eriksson et al. [20] refer to this part of ethical competence as *being* – having good character, being an ethical person. Several studies have investigated what kind of virtues a good nurse or a good physician should have [30–37]. Some of the virtues are related to the caregivers’ relationship to the patient, for example kindness, empathy and compassion. Others can be seen as a form of help for the caregiver. Day [33] explains the need for a nurse to have enough courage to do what the practice defines as good – with the risk of physical harm/injury/contagion, to stand up for good care against organizations and to sometimes delay things in order to be able to support patients and families.

Several respondents in this study also emphasized the need for knowledge about ethical theories and principles. Eriksson et al. [20] refer to this part of ethical competence as *knowing* – to have knowledge of ethical theories, and of relevant ethical guidelines. Some of the physicians could use ethical language to refer to some principles and models, using the common descriptions and names. The nurses did not describe the ethical models and principles using “ethical language” to the same extent as the physicians, but did talk about how they acted on them.

The respondents in this study used their knowledge of ethics to manage more or less severe ethical dilemmas throughout their work days. The physicians made choices in treatment together with colleagues and/or patients and relatives. They decided to inform or not inform patients and relatives about the DNR decision. They weighed ethical principles against each other to find the right decision for the individual patient. Eriksson et al. [20] refer to this component of ethical competence as *doing* – to use ethics as a guide for how to act in the best way.

In addition, virtues, in Eriksson’s et al. words “being” [20], were expressed as part of an ethical competence. That virtues are important in health care ethics has previously been reported. In a study by Aydin et al. [30], first-year nursing students had a different understanding of the virtues required for being a good nurse than fourth-year students. This could be the result of a stepwise maturation during the four-year education in the investigated program. In our study, the participants mentioned ethical dialogues and discussions on ethics on a regular basis as important methods to develop ethical competence. In line with Eriksson et al. [20] such communicative efforts could be a way to develop virtues, apart from learning through experience and being influenced by role models.

Further, the respondents mentioned education and courses, experience, self-reflection and a good ethical climate as requisites for being able to learn and develop ethical competence. This is supported in several studies, for example, Robichaux [29], who found continuing education in ethics, interdisciplinary ethics rounds and guidance from experienced colleagues to be important aspects of developing ethical competence. Lee et al. [38] found in a study of nursing students that ethics was best learned through meeting with patients and a broad perspective in teaching ethics, focusing on medicine and nursing, patient preferences, quality of life and contextual features. Lechasseur et al. [19] also found that experience is a positive factor for developing ethical competence.

Several of the interviewed physicians described how they did not inform patients or relatives of a DNR decision, and some of them felt guilty for this. However, the physicians reported their ethical consideration before making a decision that deviated from the guidelines, weighing the requirement for providing information against the risk that the patient and/or family would suffer more if they were informed of a DNR decision than if they were not informed. The phenomenon of breaking rules has been described by Kälvemark et al. [3], who found both voluntary and forced rule-breaking among nurses and physicians. They also reported how nurses and physicians justified the breaking of the rules by referring to patients’ needs, ethical considerations and their own conscience. Rejno et al. [39] found that members of medical teams working with stroke patients sometimes withheld the truth about patients close to death, in order to protect their relatives. They sometimes waited for the right time to provide the information, and did not want to add to the sorrow. By withholding the truth, they weighed the destroyed hope against compassion, and convinced themselves that the decision was morally right before acting on it.

In the present study some physicians reported feeling guilty about deviating from the rules. That was not reported in the study by Kälvemark et al. [3]. Some physicians described how they did not use the word DNR, but instead spoke of creating a calm situation around the patient and ensuring pain relief. They also described that it was likely that there was often some form of consensus with relatives about not performing CPR, since very few relatives became upset when CPR was not initiated in the event of cardiac arrest. Relatives’ understanding of the patient’s closeness to death might also increase if they were able to see the patient often and follow the different stages of the disease. Encouraging relatives to be with the dying patient may help them later in the process of grieving [40, 41].

Hence, physicians saw guidelines sometimes as an obstacle, while nurses in the present study rather described guidelines as a support. However, the results showed a quite vague knowledge on guidelines among the nurses. They could not mention them by name, but knew roughly what they said. This is in line with previous research on guidelines in clinical practice [21].

Since these interviews were conducted, a new law was passed in the Swedish parliament: the Patient Act [42]. This law further strengthens patients’ right to participation in their care, emphasizing patients’ right to be informed. If the patient is not capable of taking in information, the relatives should be informed. However, according to nurses in our previous study, there are physicians who refrain by default from informing them, due to routine at the clinic, or because they feel uncomfortable or scared in such situations [2]. This picture was strengthened by the present study, as some respondents expressed that they did not always inform in accordance with guidelines. This could be a voluntary or forced breaking of rules [3]. Rejno et al. [39] reported that one reason for not telling the truth about dying patients to their relatives was that the physician in charge did not want to be the one to break the bad news.

Overall, several respondents in this study expressed knowledge of ethical theories and principles, and also described the virtues a nurse or a physician needed to possess in order to make or be part of decisions on DNR in oncology or hematology care. They weighed ethical principles against each other, with the patients’ best interest in focus. When they withheld information, they did so after thorough ethical consideration and with the intention to not cause harm.

As ethical competence is closely connected to work tasks [15] it is not surprising that nurses and physicians differ to some extent in their answers on what ethical competence is and how it can be developed. Physicians have the medical responsibility, while nurses are responsible for providing good nursing care. Further, the physicians in our study gave richer answers regarding what ethical competence was. They were, as mentioned, also asked further questions about the subject, and they also used more “ethical language” than the nurses did. That could be explained by more education in ethics during their formal training, but it may also be an effect of more regular ethical discussions with colleagues on ethical dilemmas.

### Study limitations

The interview guide was not exactly the same for the two interview occasions. The nurses, who were interviewed first, were asked about the content of ethical competence. They had engaged in ethics discussions and ethics rounds to varying degrees, often as single events after especially difficult situations, though occasionally as repeated events on a regular basis. They referred to ethics rounds as an opportunity for the team to talk about difficult situations regarding DNR orders, and they experienced this as a source of learning more about ethics and more about the teams’ different views on the situations. Based on these answers, a question about how ethical competence can be taught and developed was added to the physicians’ interview guide. Since the nurses were not asked this question, we cannot know if they had more opinions on the topic.

There is a risk of selection bias, as the nurses and physicians who agreed to participate in the study may have an interest in the subject and have thought about the DNR process and its ethical perspectives. This can enrich the material, but it may also mean that nurses and physicians who might feel uncomfortable in DNR situations are not represented in the material.

### Clinical implications

The foundation of ethical competence is laid in the formal education of nurses and physicians, and is then further developed and consolidated in clinical practice. However, opportunities for reflection are also needed. Therefore, continued ethical education and discussions for the further development of a common ethical language are crucial. Thus, head of departments are responsible for providing a good ethical working climate that can increase ethical competence. In such dialogues, ethical guidelines could be raised and discussed, in order to make different staff categories more familiar with them and make them more aware of how they should be handled in complicated ethical dilemmas. Enhancement of ethical competence will help improve cooperation between nurses and physicians regarding patients and DNR decisions, in their efforts to act in the best interest of the patient.

## Conclusion

In order to make ethically sound DNR decisions in oncology and hematology care, physicians and nurses need all three aspects of ethical competence previously identified: *being, doing* and *knowing*. Hence, they need to develop appropriate virtues and improve their knowledge of ethical theories and relevant clinical guidelines. Furthermore, well-developed ethical competence includes the ability to act upon the ethical judgements you have made. The respondents in this study reflected ethically on their work, and if they deviated from guidelines in relation to DNR decisions, they did so after thorough ethical consideration. However, they also described how the workplace needed to create opportunities for reflection on and discussion of ethics in end of life care in oncology and hematology, in order to keep contextual ethical competence on a high level.

## Abbreviations

CPR: Cardiopulmonary resuscitation
DNR: Do-not-resuscitate
